# Supplementary material for: High Pressure and Pasteurization Effects on Dairy Cream
Source: Foods. 2023 Oct 1;12(19):3640. doi: 10.3390/foods12193640 (PMC10572675; doi:10.3390/foods12193640)
Supplement: Supplementary file 1 [file foods-12-03640-s001.zip › foods-2620745-supplementary.pdf]

Table S1: pH and colour analysis of the different treatment condition (heat, 450/5 and 600/5) of the initial cream and right after processing (0 d) and after 3 (3 d), 10 (10 d), 28 (28 d) and 52 (52 d) days of storage at 4 °C. Results are presented as mean ± standard deviation. Different letters represent statistically differences ($p<0.05$) between storage days for each condition (A-B) and between treatment conditions for each storage day (a-b).

Storage time (days)	Conditions	pH	*L**	*a**	*b**	*ΔE**
0	**Initial**	6.74 ± 0.05^{aA}	47.3 ± 0.32^{aA}	– 0.05 ± 0.11^{aA}	4.80 ± 0.32^{aA}	
	**Heat-treated**	6.93 ± 0.01^{aA}	48.5 ± 0.98^{aA}	– 0.09 ± 0.15^{aA}	4.97 ± 0.98^{aA}	1.48 ± 0.60^{aA}
	**450 MPa/5 min**	7.00 ± 0.02^{bA}	49.0 ± 0.16^{aA}	0.31 ± 0.04^{aB}	5.04 ± 0.16^{aA}	2.19 ± 2.23^{aA}
	**600 MPa/5 min**	6.86 ± 0.03^{abA}	48.6 ± 0.08^{aA}	0.19 ± 0.04^{aB}	5.19 ± 0.08^{aA}	1.43 ± 1.00^{aA}
5	**Heat-treated**	6.83 ± 0.02^{aA}	50.9 ± 2.20^{aA}	0.11 ± 0.08^{abA}	6.67 ± 0.40^{bA}	4.12 ± 0.87^{aA}
	**450 MPa/5 min**	6.95 ± 0.03^{bA}	49.5 ± 0.85^{aA}	0.51 ± 0.02^{abB}	6.11 ± 0.16^{abA}	2.87 ± 2.26^{aA}
	**600 MPa/5 min**	7.06 ± 0.12^{bA}	51.5 ± 0.81^{abA}	0.47 ± 0.01^{bB}	6.58 ± 0.13^{bA}	4.85 ± 2.68^{aA}
9	**Heat-treated**	6.89 ± 0.35^{aA}	52.1 ± 1.70^{aA}	0.15 ± 0.08^{bA}	6.83 ± 0.30^{bA}	5.29 ± 1.77^{aA}
	**450 MPa/5 min**	6.94 ± 0.20^{bA}	51.4 ± 0.33^{aA}	0.60 ± 0.02^{bB}	6.58 ± 0.08^{bA}	4.64 ± 1.98^{aA}
	**600 MPa/5 min**	6.86 ± 0.20^{abA}	53.5 ± 2.37^{abA}	0.53 ± 0.03^{bB}	6.91 ± 0.32^{bA}	6.66 ± 1.58^{aA}
18	**Heat-treated**	6.85 ± 0.18^{aA}	52.7 ± 0.96^{aA}	0.17 ± 0.04^{bA}	6.92 ± 0.20^{bA}	5.88 ± 2.86^{aA}
	**450 MPa/5 min**	6.66 ± 0.12^{aA}	52.0 ± 0.75^{aA}	0.56 ± 0.04^{bB}	6.64 ± 0.08^{bA}	5.20 ± 1.55^{aA}
	**600 MPa/5 min**	6.67 ± 0.05^{aA}	53.3 ± 2.15^{abA}	0.41 ± 0.03^{abB}	6.88 ± 0.24^{bA}	6.40 ± 1.01^{aA}
33	**Heat-treated**	6.64 ± 0.05^{aA}	51.4 ± 0.96^{aA}	0.04 ± 0.03^{abA}	6.70 ± 0.13^{bA}	4.86 ± 2.51^{aA}
	**450 MPa/5 min**	–	–	–	–	–
	**600 MPa/5 min**	6.66 ± 0.02^{aA}	53.4 ± 1.40^{abA}	0.35 ± 0.07^{abB}	7.07 ± 0.24^{bA}	6.62 ± 1.00^{aA}
51	**Heat-treated**	6.69 ± 0.22^{aA}	53.6 ± 2.00^{aA}	0.17 ± 0.09^{bA}	7.26 ± 0.36^{bA}	8.01 ± 3.71^{aA}
	**450 MPa/5 min**	–	–	–	–	–
	**600 MPa/5 min**	6.62 ± 0.05^{aA}	55.2 ± 0.39^{bA}	0.54 ± 0.01^{bB}	7.45 ± 0.04^{bA}	8.43 ± 2.60^{aA}

Table S2: pH and colour analysis of the different treatment condition (heat, 600/5 and 600/15) of the initial cream and right after processing (0 d) and after 3 (3 d), 10 (10 d), 28 (28 d) and 52 (52 d) days of storage at 4 °C. Results are presented as mean ± standard deviation. Different letters represent statistically differences ($p<0.05$) between storage days for each condition (A-C) and between treatment conditions for each storage day (a-b).

Storage time (days)	Conditions	pH	*L**	*a**	*b**	*ΔE**
0	**Initial**	6.91 ± 0.14^{aA}	51.0 ± 0.27^{aA}	0.42 ± 0.05^{aC}	6.16 ± 0.27^{aA}	
	**Heat-treated**	6.75 ± 0.01^{bA}	50.2 ± 0.12^{aA}	-0.08 ± 0.00^{aA}	6.15 ± 0.12^{aA}	1.40 ± 1.08^{aA}
	**600 MPa/15 min**	6.88 ± 0.05^{bA}	50.7 ± 0.16^{aA}	0.25 ± 0.02^{abB}	6.63 ± 0.16^{aA}	2.51 ± 1.49^{aA}
3	**Heat-treated**	6.66 ± 0.07^{bA}	54.1 ± 2.06^{aA}	0.12 ± 0.08^{bA}	7.08 ± 0.40^{bA}	4.72 ± 2.15^{aA}
	**600 MPa/15 min**	6.92 ± 0.08^{bB}	52.0 ± 0.48^{abA}	0.33 ± 0.06^{bB}	6.65 ± 0.05^{aA}	1.72 ± 0.91^{aA}
10	**Heat-treated**	6.44 ± 0.01^{aA}	52.4 ± 0.97^{aA}	0.06 ± 0.03^{abA}	6.80 ± 0.24^{abA}	3.09 ± 1.31^{aA}
	**600 MPa/15 min**	6.60 ± 0.10^{aA}	52.0 ± 1.03^{abA}	0.35 ± 0.03^{bB}	6.79 ± 0.19^{aA}	2.94 ± 1.32^{aA}
28	**Heat-treated**	6.66 ± 0.16^{bA}	53.8 ± 1.50^{aA}	0.16 ± 0.05^{bA}	7.09 ± 0.26^{bA}	4.15 ± 1.57^{aA}
	**600 MPa/15 min**	6.54 ± 0.03^{aA}	56.5 ± 3.11^{bA}	0.38 ± 0.05^{bB}	7.45 ± 0.40^{aA}	5.79 ± 4.78^{aA}
52	**Heat-treated**	–	–	–	–	–
	**600 MPa/15 min**	6.62 ± 0.02^{aA}	53.6 ± 0.54^{abA}	0.13 ± 0.03^{aA}	7.14 ± 0.04^{aA}	3.01 ± 2.00^{aA}

Table S3: Cream fatty acids composition (% of total fatty acids), at different treatment condition (heat, 600/5 and 600/15) of the initial cream and right after processing (0 d) and after 3 (3 d), 10 (10 d), 28 (28 d) and 52 (52 d) days of storage at 4 °C. Results are presented as mean ± standard deviation. Different letters represent statistically differences ($p<0.05$) between storage days for each condition (A-B) and between treatment conditions for each storage day (a-b).

	Day 0				Day 3			Day 10			Day 28			Day 52		
Fatty acids	Initial	Heat-treated	600 MPa/5 min	600 MPa/15 min	Heat-treated	600 MPa/5 min	600 MPa/15 min	Heat-treated	600 MPa/5 min	600 MPa/15 min	Heat-treated	600 MPa/5 min	600 MPa/15 min	Heat-treated	600 MPa/5 min	600 MPa/15 min
C4:0	3.47 ± 0.05^{aA}	3.01 ± 0.42^{aA}	3.29 ± 0.23^{aA}	3.40 ± 0.03^{aA}	3.19 ± 0.09^{aA}	3.26 ± 0.17^{aA}	3.12 ± 0.13^{aA}	3.15 ± 0.26^{aA}	2.89 ±0.66^{aA}	2.75 ± 0.36^{aA}	2.55 ± 0.02^{aA}	3.13 ± 0.21^{aA}	3.40 ± 0.02^{aA}	3.25 ± 0.04^{aA}	3.10 ± 0.03^{aA}	2.97 ± 0.03^{aA}
C6:0	2.78 ± 0.01^{aA}	2.56 ± 0.10^{aA}	2.69 ± 0.03^{aA}	2.64 ± 0.02^{aA}	2.68 ± 0.02^{aA}	2.72 ± 0.05^{aA}	2.72 ± 0.05^{aA}	2.73 ± 0.06^{aA}	2.69 ± 0.20^{aA}	2.55 ± 0.24^{aA}	2.38 ± 0.17^{aA}	2.56 ± 0.10^{aA}	2.65 ± 0.01^{aA}	2.61 ± 0.01^{aA}	2.60 ± 0.02^{aA}	2.59 ± 0.02^{aA}
C8:0	1.55 ± 0.01^{aA}	1.46 ± 0.02^{abA}	1.50 ± 0.02^{aA}	1.46 ± 0.02^{aA}	1.50 ± 0.01^{abA}	1.52 ± 0.02^{aA}	1.55 ± 0.01^{aA}	1.54 ± 0.02^{bA}	1.55 ± 0.07^{aA}	1.47 ±0.10^{aA}	1.38 ± 0.10^{aA}	1.44 ± 0.04^{aA}	1.47 ± 0.01^{aA}	1.46 ± 0.00^{abA}	1.46 ± 0.01^{aA}	1.47 ± 0.01^{aA}
C10:0	3.35 ± 0.02^{aA}	3.16 ± 0.02^{aA}	3.19 ± 0.04^{aA}	3.12 ± 0.04^{aA}	3.23 ± 0.02^{aA}	3.26 ± 0.05^{aA}	3.31 ± 0.03^{aA}	3.29 ± 0.03^{aA}	3.32 ± 0.11^{aA}	3.23 ± 0.17^{aA}	3.05 ± 0.14^{aA}	3.15 ± 0.02^{aA}	3.15 ± 0.01^{aA}	3.15 ± 0.01^{aA}	3.14 ± 0.04^{aA}	3.16 ± 0.01^{aA}
C10:1	0.39 ± 0.00^{aA}	0.37 ± 0.00^{abA}	0.38 ± 0.01^{aA}	0.37 ± 0.00^{aA}	0.38 ±0.00^{abA}	0.39 ± 0.01^{aA}	0.40 ± 0.01^{aA}	0.39 ± 0.00^{bA}	0.40 ± 0.01^{aA}	0.39 ± 0.02^{aA}	0.36 ± 0.02^{aA}	0.38 ± 0.00^{aA}	0.38 ± 0.00^{aA}	0.37 ± 0.00^{abA}	0.37 ± 0.00^{aA}	0.37 ± 0.00^{aA}
C12:0	4.04 ± 0.03^{aB}	3.73 ± 0.01^{aA}	3.74 ± 0.04^{aA}	3.69 ± 0.03^{aA}	3.78 ± 0.03^{aA}	3.80 ± 0.05^{aA}	3.83 ± 0.02^{abA}	3.82 ± 0.04^{aA}	3.87 ± 0.07^{aA}	3.91 ± 0.16^{bA}	3.68 ± 0.07^{aA}	3.76 ± 0.05^{aA}	3.72 ± 0.00^{abA}	3.72 ± 0.00^{aA}	3.72 ± 0.04^{aA}	3.75 ± 0.02^{abA}
C14:0	11.25 ± 0.07^{aA}	11.16 ± 0.04^{aA}	11.13 ± 0.05^{aA}	11.14 ± 0.06^{aA}	11.21 ± 0.03^{aA}	11.25 ± 0.12^{aA}	11.32 ± 0.01^{aA}	11.29 ± 0.13^{aA}	11.42 ± 0.12^{aAB}	11.74 ± 0.31^{bB}	11.08 ± 0.05^{aA}	11.33 ± 0.20^{aA}	11.19 ± 0.00^{aA}	11.24 ± 0.02^{aA}	11.18 ± 0.08^{aA}	11.28 ± 0.04^{aA}
C14:1t	0.35 ± 0.01^{aA}	0.37 ± 0.03^{aA}	0.33 ± 0.07^{aA}	0.22 ± 0.12^{aA}	0.34 ± 0.03^{aA}	0.31 ± 0.07^{aA}	0.38 ± 0.02^{aA}	0.37 ± 0.01^{aA}	0.28 ± 0.08^{aA}	0.37 ± 0.01^{aA}	0.37 ± 0.02^{aA}	0.27 ± 0.14^{aA}	0.35 ± 0.01^{aA}	0.20 ± 0.12^{aA}	0.35 ± 0.01^{aA}	0.34 ± 0.01^{aA}
C14:1	1.23 ± 0.00^{aA}	1.25 ± 0.01^{aA}	1.25 ± 0.01^{aA}	1.24 ± 0.01^{aA}	1.28 ± 0.02^{aA}	1.25 ± 0.02^{aA}	1.27 ± 0.01^{abA}	1.26 ± 0.01^{aA}	1.26 ± 0.01^{aA}	1.31 ± 0.03^{bA}	1.24 ± 0.00^{aA}	1.25 ± 0.02^{aA}	1.24 ± 0.00^{aA}	1.24 ± 0.00^{aA}	1.23 ± 0.01^{aA}	1.23 ± 0.00^{aA}
ai-C15:0	0.66 ± 0.00^{aA}	0.73 ± 0.01^{abB}	0.73 ± 0.00^{aB}	0.72 ± 0.00^{aB}	0.76 ± 0.02^{bB}	0.72 ± 0.01^{aA}	0.73 ± 0.00^{abAB}	0.73 ± 0.01^{abA}	0.73 ± 0.00^{aA}	0.75 ± 0.01^{bA}	0.73 ± 0.00^{abA}	0.73 ± 0.01^{aA}	0.72 ± 0.00^{abA}	0.72 ± 0.00^{aA}	0.72 ± 0.00^{aA}	0.72 ± 0.00^{aA}
C15:0	1.07 ± 0.00^{aA}	1.10 ± 0.01^{aA}	1.09 ± 0.01^{aA}	1.09 ± 0.01^{aA}	1.10 ± 0.00^{aA}	1.10 ± 0.01^{aA}	1.10 ± 0.00^{aA}	1.10 ± 0.01^{aA}	1.11 ± 0.00^{aAB}	1.14 ± 0.02^{bB}	1.11 ± 0.01^{aA}	1.11 ± 0.01^{aA}	1.09 ± 0.00^{aA}	1.10 ± 0.01^{aA}	1.10 ± 0.01^{aA}	1.10 ± 0.00^{aA}
i-C16:0	0.33 ± 0.01^{aA}	0.36 ± 0.01^{aB}	0.36 ± 0.00^{aB}	0.35 ± 0.00^{aB}	0.37 ± 0.01^{aB}	0.36 ± 0.01^{aAB}	0.35 ± 0.00^{aA}	0.36 ± 0.00^{aA}	0.36 ± 0.00^{aA}	0.37 ± 0.01aA	0.36 ± 0.00^{aA}	0.36 ± 0.00^{aA}	0.35 ± 0.00^{aA}	0.35 ± 0.00^{aA}	0.36 ± 0.00^{aA}	0.36 ± 0.00^{aA}
C16:0	24.50 ± 0.12^{aB}	23.74 ± 0.13^{aA}	23.66 ± 0.06^{aA}	23.73 ± 0.10^{abA}	23.63 ± 0.08^{aA}	23.77 ± 0.14^{aA}	23.70 ± 0.05^{aA}	23.75 ± 0.23^{aA}	24.00 ± 0.13^{aA}	24.20 ± 0.25^{bA}	24.06 ± 0.15^{aA}	23.92 ± 0.16^{aA}	23.76 ± 0.01^{abA}	23.84 ± 0.08^{aA}	23.82 ± 0.07^{aA}	23.92 ± 0.05^{abA}
C16:1T	0.52 ± 0.01^{aA}	0.55 ± 0.03^{aA}	0.54 ± 0.00^{aA}	0.54 ± 0.02^{aA}	0.54 ± 0.02^{aA}	0.53 ± 0.04^{aA}	0.56 ± 0.03^{aA}	0.55 ± 0.02^{aA}	0.55 ± 0.02^{aA}	0.54 ± 0.02^{aA}	0.56 ± 0.02^{aA}	0.54 ± 0.02^{aA}	0.55 ± 0.02^{aA}	0.52 ± 0.03^{aA}	0.54 ± 0.04^{aA}	0.52 ± 0.01^{aA}
C16:1C	2.75 ± 0.02^{aB}	2.63 ± 0.03^{aA}	2.62 ± 0.00^{aA}	2.63 ± 0.01^{aA}	2.70 ± 0.06^{aA}	2.61 ± 0.02^{aA}	2.63 ± 0.01^{aA}	2.63 ± 0.03^{aA}	2.63 ± 0.02^{aA}	2.67 ± 0.01^{aA}	2.65 ± 0.03^{aA}	2.63 ± 0.02^{aA}	2.61 ± 0.00^{aA}	2.66 ± 0.07^{aA}	2.62 ± 0.01^{aA}	2.61 ± 0.01^{aA}
ai-C17:0	0.83 ± 0.01^{aA}	0.87 ± 0.01^{aB}	0.87 ± 0.01^{aB}	0.88 ± 0.01^{aB}	0.87 ± 0.01^{aA}	0.87 ± 0.01^{aA}	0.87 ± 0.01^{aA}	0.88 ± 0.01^{aA}	0.87 ± 0.01^{aA}	0.88 ± 0.01^{aA}	0.88 ± 0.01^{aA}	0.87 ± 0.01^{aA}	0.87 ± 0.00^{aA}	0.87 ± 0.00^{aA}	0.87 ± 0.00^{aA}	0.87 ± 0.00^{aA}
C17:0	0.63 ± 0.00^{aA}	0.64 ± 0.00^{aA}	0.63 ± 0.00^{aA}	0.65 ± 0.01^{aA}	0.66 ± 0.01^{aA}	0.64 ± 0.00^{aA}	0.64 ± 0.01^{aA}	0.64 ± 0.01^{aA}	0.64 ± 0.01^{aA}	0.65 ± 0.03^{aA}	0.66 ± 0.02^{aA}	0.65 ± 0.01^{aA}	0.64 ± 0.00^{aA}	0.65 ± 0.01^{aA}	0.64 ± 0.01^{aA}	0.65 ± 0.00^{aA}
i-C18:0	0.32 ± 0.02^{aA}	0.31 ± 0.00^{aA}	0.31 ± 0.01^{aA}	0.31 ± 0.00^{aA}	0.31 ± 0.01^{aA}	0.31 ± 0.01^{aA}	0.32 ± 0.01^{aA}	0.33 ± 0.02^{aA}	0.31 ± 0.00^{aA}	0.31 ± 0.01^{aA}	0.32 ± 0.00^{aA}	0.32 ± 0.01^{aA}	0.31 ± 0.00^{aA}	0.31 ± 0.00^{aA}	0.31 ± 0.00^{aA}	0.31 ± 0.00^{aA}
C18:0	9.26 ± 0.05^{aA}	9.91 ± 0.06^{abB}	9.79 ± 0.06^{aB}	9.91 ± 0.08^{aB}	9.79 ± 0.04^{abA}	9.76 ± 0.11^{aA}	9.68 ± 0.06^{aA}	9.64 ± 0.09^{aA}	9.80 ± 0.22^{aA}	9.61 ± 0.33^{aA}	10.14 ± 0.12^{bA}	9.88 ± 0.09^{aA}	9.89 ± 0.02^{aA}	9.95 ± 0.04^{abA}	9.99 ± 0.01^{aA}	10.04 ± 0.03^{aA}
C18:1t	4.05 ± 0.02^{aA}	4.65 ± 0.06^{aB}	4.62 ± 0.03^{aB}	4.64 ± 0.04^{aB}	4.64 ± 0.02^{aA}	4.53 ± 0.03^{aA}	4.59 ± 0.01^{aA}	4.56 ± 0.05^{aA}	4.54 ± 0.09^{aA}	4.55 ± 0.13^{aA}	4.75 ± 0.06^{aA}	4.63 ± 0.05^{aA}	4.59 ± 0.01^{aA}	4.65 ± 0.03^{aA}	4.65 ± 0.05^{aA}	4.66 ± 0.02^{aA}
C18:1c	20.03 ± 0.11^{aA}	20.82 ± 0.15^{aA}	20.70 ± 0.12^{aA}	20.81 ± 0.05^{aA}	20.45 ± 0.09^{aA}	20.32 ± 0.17^{aA}	20.39 ± 0.10^{aA}	20.38 ± 0.21^{aA}	20.36 ± 0.43^{aA}	20.24 ± 0.58^{aA}	20.96 ± 0.23^{aA}	20.56 ± 0.19^{aA}	20.56 ± 0.03^{aA}	20.75 ± 0.04^{aA}	20.76 ± 0.08^{aA}	20.63 ± 0.09^{aA}
C18:2t	0.83 ± 0.07^{aA}	0.86 ± 0.04 ^{aA}	0.85 ± 0.03 ^{aA}	0.76 ± 0.14^{aA}	0.85 ± 0.08^{aA}	0.84 ± 0.06^{aA}	0.81 ± 0.04^{aA}	0.89 ± 0.08^{aA}	0.79 ± 0.04^{aA}	0.84 ± 0.02^{aA}	0.84 ± 0.09^{aA}	0.86 ± 0.02^{aA}	0.84 ± 0.02^{aA}	0.84 ± 0.03^{aA}	0.86 ± 0.02^{aA}	0.89 ± 0.05^{aA}
C18:2c	2.99 ± 0.05^{aA}	2.95 ± 0.01^{aA}	2.96 ± 0.01^{aA}	2.96 ± 0.01^{aA}	2.91 ± 0.01^{aA}	2.91 ± 0.03^{aA}	2.90 ± 0.01^{aA}	2.90 ± 0.02^{aA}	2.90 ± 0.06^{aA}	2.93 ± 0.10^{aA}	3.00 ± 0.03^{aA}	2.93 ± 0.02^{aA}	2.93 ± 0.01^{aA}	2.93 ± 0.03^{aA}	2.97 ± 0.04^{aA}	2.93 ± 0.01^{aA}
C18:3n-6	0.06 ± 0.01^{aA}	0.07 ± 0.01^{aA}	0.07 ± 0.01^{aA}	0.07 ± 0.01^{aA}	0.06 ± 0.02^{aA}	0.07 ± 0.00^{aA}	0.08 ± 0.01^{aA}	0.08 ± 0.01^{aA}	0.07 ± 0.00^{aA}	0.08 ± 0.01^{aA}	0.07 ± 0.01^{aA}	0.07 ± 0.00^{aA}	0.07 ± 0.00^{aA}	0.08 ± 0.00^{aA}	0.08 ± 0.01^{aA}	0.07 ± 0.01^{aA}
C18:3n-3	0.33 ± 0.00^{aA}	0.28 ± 0.00^{aA}	0.28 ± 0.01^{aA}	0.24 ± 0.07^{aA}	0.29 ± 0.00^{aA}	0.28 ± 0.00^{aA}	0.28 ± 0.00^{aA}	0.27 ± 0.00^{aA}	0.28 ± 0.01^{aA}	0.27 ± 0.01^{aA}	0.29 ± 0.00^{aA}	0.28 ± 0.01^{aA}	0.28 ± 0.00^{aA}	0.29 ± 0.01^{aA}	0.28 ± 0.00^{aA}	0.28 ± 0.00^{aA}
C20:0	0.19 ± 0.01^{aA}	0.21 ± 0.00^{aA}	0.21 ± 0.01^{aA}	0.22 ± 0.02^{aA}	0.22 ± 0.01^{aA}	0.20 ±0.01^{aA}	0.21 ± 0.01^{aA}	0.20 ± 0.00^{aA}	0.20 ± 0.01^{aA}	0.20 ± 0.01^{aA}	0.21 ± 0.00^{aA}	0.21 ± 0.00^{aA}	0.21 ± 0.00^{aA}	0.23 ± 0.02^{aA}	0.21 ± 0.00^{aA}	0.22 ± 0.01^{aA}
C20:1	0.22 ± 0.00^{aA}	0.24 ± 0.01^{bAB}	0.24 ± 0.00^{bAB}	0.25 ± 0.02^{bB}	0.25 ± 0.01^{bA}	0.24 ± 0.01^{bA}	0.23 ± 0.01^{bA}	0.23 ± 0.01^{bA}	0.23 ± 0.01^{bA}	0.23 ± 0.01^{bA}	0.25 ± 0.01^{bA}	0.24 ± 0.01^{bA}	0.23 ± 0.00^{bA}	0.16 ± 0.00^{aA}	0.17 ± 0.00^{aA}	0.17 ± 0.01^{aA}
C22:0	0.06 ± 0.00^{aA}	0.07 ± 0.00^{aA}	0.07 ± 0.00 ^{aA}	0.07 ± 0.00^{aA}	0.06 ± 0.02aA	0.07 ± 0.00aA	0.06 ± 0.00^{aA}	0.06 ± 0.00^{aA}	0.06 ± 0.00^{aA}	0.07 ± 0.00^{aA}	0.07 ± 0.00^{aA}	0.07 ± 0.00aA	0.07 ± 0.00aA	0.29 ± 0.00^{bA}	0.30 ± 0.00^{bA}	0.30 ± 0.00^{bA}
C24:0	0.15 ± 0.02^{aA}	0.10 ± 0.00^{aA}	0.10 ± 0.01^{aA}	0.11 ± 0.01^{aA}	0.16 ± 0.03^{bA}	0.14 ± 0.03^{aA}	0.12 ± 0.01^{aA}	0.11 ± 0.01^{abA}	0.08 ± 0.00^{aA}	0.09 ± 0.01^{aA}	0.10 ± 0.00^{aA}	0.10 ± 0.01^{aA}	0.10 ± 0.01^{aA}	0.10 ± 0.01^{aA}	0.11 ± 0.01^{aA}	0.11 ± 0.02^{aA}
Saturated	64.60 ± 0.31^{aB}	63.26 ± 0.37^{aAB}	63.50 ± 0.19^{aAB}	63.65 ± 0.22^{aA}	63.61 ± 0.16^{aA}	63.96 ± 0.43^{aA}	63.78 ± 0.12^{aA}	63.76 ± 0.07^{aA}	64.04 ± 0.81^{aA}	64.06 ± 0.78^{aA}	62.91 ± 0.22^{aA}	63.75 ± 0.14^{aA}	63.75 ± 0.02^{aA}	64.12 ± 0.16^{aA}	63.88 ± 0.27^{aA}	64.07 ± 0.08^{aA}
Monounsaturated	25.05 ± 0.11^{aA}	25.79 ± 0.21^{aA}	25.64 ± 0.12^{aA}	25.74 ± 0.06^{aA}	25.37 ± 0.10^{aA}	25.32 ± 0.18^{aA}	25.44 ± 0.04^{aA}	25.33 ± 0.22^{aA}	25.32 ± 0.45^{aA}	25.26 ± 0.54^{aA}	25.91 ± 0.25^{aA}	25.52 ± 0.18^{aA}	25.46 ± 0.02^{aA}	25.63 ± 0.10^{aA}	25.61 ± 0.09^{aA}	25.47 ± 0.09^{aA}
Polyunsaturated	4.35 ± 0.13^{aA}	4.23 ± 0.06^{bA}	4.23 ± 0.07^{bA}	4.18 ± 0.05^{bA}	4.36 ± 0.18^{bA}	4.24 ± 0.17^{bA}	4.12 ± 0.03^{bA}	4.14 ± 0.05^{bA}	4.20 ± 0.19^{bA}	4.10 ± 0.14^{bA}	4.37 ± 0.03^{bA}	4.17 ± 0.06^{bA}	4.16 ± 0.02^{bA}	3.73 ± 0.03^{aA}	3.77 ± 0.06^{aA}	3.72 ± 0.01^{aA}
